# Decreased extracellular pH inhibits osteogenesis through proton-sensing GPR4-mediated suppression of yes-associated protein

**DOI:** 10.1038/srep26835

**Published:** 2016-06-03

**Authors:** Shi-Cong Tao, You-Shui Gao, Hong-Yi Zhu, Jun-Hui Yin, Yi-Xuan Chen, Yue-Lei Zhang, Shang-Chun Guo, Chang-Qing Zhang

**Affiliations:** 1 Department of Orthopedic Surgery, Shanghai Jiao Tong University Affiliated Sixth People’s Hospital, 600 Yishan Road, Shanghai 200233, China; 3 Institute of Microsurgery on Extremities, Shanghai Jiao Tong University Affiliated Sixth People’s Hospital, 600 Yishan Road, Shanghai 200233, China

## Abstract

The pH of extracellular fluids is a basic property of the tissue microenvironment and is normally maintained at 7.40 ± 0.05 in humans. Many pathological circumstances, such as ischemia, inflammation, and tumorigenesis, result in the reduction of extracellular pH in the affected tissues. In this study, we reported that the osteogenic differentiation of BMSCs was significantly inhibited by decreases in the extracellular pH. Moreover, we demonstrated that proton-sensing GPR4 signaling mediated the proton-induced inhibitory effects on the osteogenesis of BMSCs. Additionally, we found that YAP was the downstream effector of GPR4 signaling. Our findings revealed that the extracellular pH modulates the osteogenic responses of BMSCs by regulating the proton-sensing GPR4-YAP pathway.

The microenvironment plays important roles in the maintenance of normal tissue homeostasis and the development of diseases^1^,^2^. The proton concentration or pH of extracellular fluids (ECFs) is a basic property of the tissue microenvironment and is normally maintained at 7.40 ± 0.05. Imbalances in the extracellular pH have strong influences on the functions of organisms. Inflammation, ischemia and the microenvironments of solid tumors are often accompanied by extracellular pH (acidosis) reductions that may result in inhibited immune function^3^, enhanced normal cell necroptosis^4^, and increased tumor invasion^5^. Magnesium implants have been found to prevent bacterial biofilm formation by generating an alkaline environment^6^.

Osteomyelitis, avascular necrosis of the femoral head, and bone metastases from tumors represent bone tissue inflammation, ischemia and tumor metastasis, respectively, and all of these conditions induce acidic microenvironments and severe bone destruction^7^,^8^,^9^. Magnesium implants are able to stimulate new bone formation by enhancing the osteogenic activities of bone marrow-derived mesenchymal stem cells (BMSCs)^10^,^11^. We hypothesized that alterations in the extracellular pH might be an important mechanism that leads to changes in cellular osteogenic responses and bone tissue growth.

The molecular mechanisms by which cells respond to extracellular pH changes are not fully understood. A group of G-protein-coupled receptors (GPCRs), including GPR4^12^, GPR65 (TDAG8)^13^, GPR68 (OGR1)^14^ and GPR132 (G2A)^15^, have been identified as proton-sensing machineries that can be activated with increases in the proton concentration. GPR68 is usually coupled with Gq/11 and activates phospholipase C (PLC)/Ca^2+^ signaling, and GPR4, GPR65 and GPR132 typically activate the adenylyl cyclase/cAMP/PKA pathway through Gs proteins^14^,^16^. All of these GPCRs can also induce the activation of Rho signaling via G_12/13_ 14,16. Yes-associated protein (YAP) is a major downstream effector of the Hippo pathway and partners with TEAD family transcription factors to stimulate the expression of genes that promote proliferation and inhibit apoptosis^17^. A study by Yu and colleagues^18^ revealed that YAP can be activated by G12/13- and Gq/11-coupled receptors and inhibited by Gs-coupled receptors. More recently, we found that YAP is the downstream effector of GPR68-Rho signaling and that the extracellular pH can modulate the proliferation and apoptosis of BMSCs via the regulation of the GPR68-Rho-YAP pathway^19^.

In the present study, we found that the osteogenic activities of BMSCs were decreased with reductions in the extracellular pH and that GPR4-induced suppression of YAP might be an important mechanism by which proton-induced anti-osteogenic effects are elicited in BMSCs because these effects could be blocked by the inhibition of GPR4 or the activation of YAP. To the best of our knowledge, this study is the first to demonstrate the inhibitory effects of protons on the osteogenesis of BMSCs and elucidate the underlying mechanism.

## Results

### Low extracellular pH inhibited the osteogenic differentiation of BMSCs

**Figure 1:**
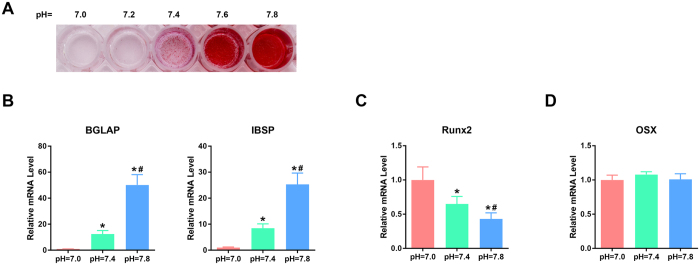
Low extracellular pH inhibits the osteogenic differentiation of BMSCs. BMSCs were cultured in osteogenic media at different pHs for 21 days, and the calcium deposits in the differentiated BMSCs were then assessed by Alizarin red S staining **(A)**. The expressions of osteogenesis-related marker genes, including BGLAP and IBSP **(B)**, Runx2 **(C)**, and Osx **(D)** were detected by qRT-PCR analyses. *P < 0.05 compared with the pH = 7.0 group; ^**#**^*P* < 0.05 compared with the pH = 7.4 group.

To explore the effects of extracellular pH on the osteogenic differentiation of BMSCs, the cells were cultured in osteogenic medium with different proton concentrations (pHs), and alizarin red S staining was performed after 21 days of differentiation. As illustrated in Fig. 1A, calcium mineral deposition in the differentiated BMSCs was significantly inhibited following incubation in a reduced pH osteogenic medium. Moreover, qRT-PCR analyses were used to detect the expressions of several osteogenesis-related marker genes, including integrin-binding sialoprotein (IBSP), bone gamma-carboxyglutamate (gla) protein (BGLAP), and osterix (Osx) on day 21 and runt-related transcription factor 2 (Runx2) on day 7. The results revealed that the reduction of the proton concentration resulted in prominent increases in the expressions of BGLAP and IBSP (Fig. 1B), which are mainly expressed during late-stage osteogenic differentiation and mineralization^20^,^21^; this latter phenomenon was also proved by the data in our study (Fig. S1). However, a lower pH microenvironment was beneficial to the expression of Runx2 (Fig. 1C), which is a bone marker that is expressed in early stage osteogenesis^22^. The changes in extracellular pH did not alter the level of the early stage osteogenic differentiation marker osterix (Osx) (Fig. 1D). These data revealed that increases in proton concentration inhibited late-stage osteogenesis in BMSCs.

### Proton-induced osteogenesis inhibition was mediated by the suppression of YAP signaling

**Figure 2:**
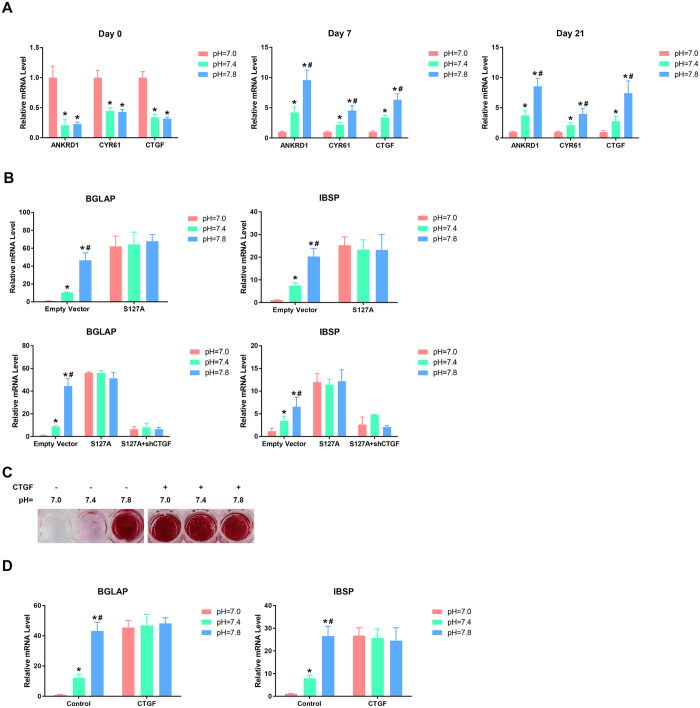
Proton-induced osteogenesis inhibition is mediated by the suppression of YAP signaling. BMSCs were incubated in osteogenic media at different pHs for 0, 7, or 21 days and then transferred to basal culture media at different pHs and cultured for 24 hours. The expressions of YAP target genes, including ANKRD1, Cyr61, and CTGF, were then assessed by qRT-PCR **(A,B)** S127A overexpression blocked the proton-induced inhibition of the expressions of IBSP and BGLAP, but this effect was markedly abolished by CTGF inhibition, as determined by qRT-PCR. The recombinant CTGF protein reversed the proton-induced reductions in calcium deposits **(C)** and the expressions of IBSP and BGLAP **(D)** in the differentiated BMSCs. *P < 0.05 compared with the pH = 7.0 group; ^**#**^*P* < 0.05 compared with the pH = 7.4 group.

Next, we investigated whether YAP was involved in the pH-dependent regulation of the osteogenesis of BMSCs. After 0, 7, or 21 days of osteogenic induction, the cells were transferred to basal culture media with different pHs and cultured for 24 hours. Next, qRT-PCR analyses were performed to assess the levels of ANKRD1, Cyr61, and CTGF, which are well-characterized downstream targets of YAP signaling^23^. As illustrated in Fig. 2A, in the un-induced BMSCs, the expression of these YAP target genes were markedly enhanced after the cells were cultured in lower-pH media. Surprisingly, following osteogenic differentiation for 7 and 21 days, the proton-mediated stimulatory effect the YAP downstream targets was significantly reversed, and their expressions were inhibited with decreases in the extracellular pH, which suggests that the signaling molecules that mediated the proton-induced YAP regulation were altered following the differentiation of the BMSCs into osteoblasts (OBs). To confirm CTGF as YAP target gene in BMSCs-derived OBs, two YAP shRNAs (shYAP #1 and shYAP #2) were used to knockdown YAP in the BMSCs and the inhibitory efficiency of these shRNAs was verified by qRT-PCR (Fig. S2A). The YAP-silenced BMSCs by shYAP #1 were induced by osteogenic media for 7 days, then transferred to basal culture media with different pHs and cultured for 24 hours. Western blotting was performed to assess the level of CTGF and the result showed that CTGF production was markedly blocked after YAP inhibition (Fig. S2B), indicating that CTGF is the downstream target of YAP in BMSCs- derived OBs.

To verify whether YAP was the critical mediator of the pH-dependent modulation of the osteogenic differentiation of BMSCs, we overexpressed S127A, which is a mutant of YAP that generates a constitutively active YAP protein that localizes to the nucleus^24^, in the BMSCs. After 21 days of induction, we detected the mRNA levels of late osteogenic differentiation markers (including BGLAP and IBSP) and found that the low pH-induced inhibition of their expressions were significantly abolished by S127A overexpression (Fig. 2B), which indicates that the proton-induced suppression of BMSC osteogenesis was attributable to YAP inhibition. Moreover, we used lentiviral shRNA to knockdown the level of CTGF, the target gene of YAP, in the YAP-S127A overexpressed BMSCs and confirmed the inhibitory efficiency of the shRNA (Fig. S3). We found that the YAP-S127A-induced up-regulation of BGLAP and IBSP in low pH conditions was blocked by CTGF inhibition (Fig. 2B), indicating that the YAP-induced osteogenesis of BMSCs was mediated by activating CTGF. Additionally, we treated the BMSCs with a recombinant CTGF protein to mimic the activation of YAP signaling and found that this protein markedly reversed the proton-induced reduction in calcium deposition and the expressions of BGLAP and IBSP in the differentiated BMSCs (Fig. 2C,D), which further demonstrated that the inactivation of YAP signaling resulted in proton-induced suppression of BMSC osteogenesis.

### GPR4 mediated the proton-induced inhibition of osteogenesis and YAP inactivation

**Figure 3:**
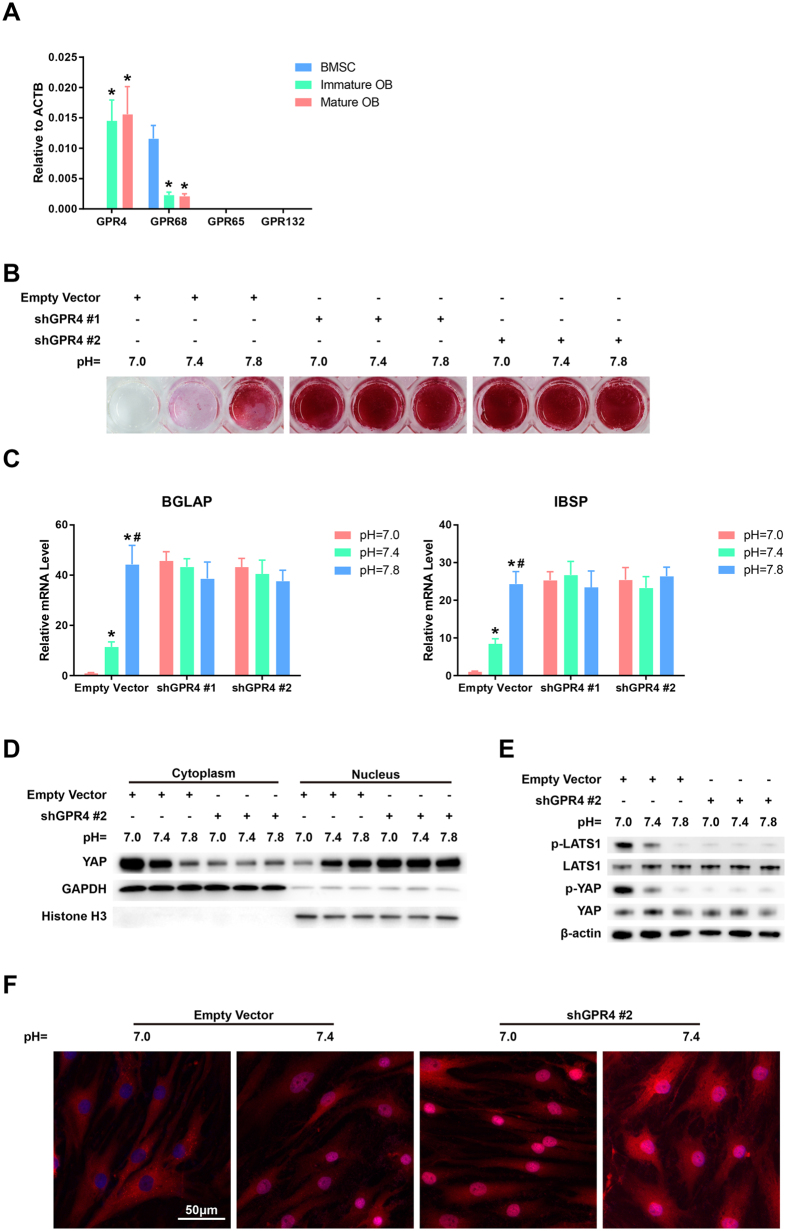
GPR4 mediates proton-induced osteogenesis inhibition and YAP inactivation. **(A)** The differential expressions of different the GPCRs in the BMSCs and their derived OBs. *P < 0.05 compared with the BMSC group. Knockdown of GPR4 abolished the proton-induced inhibitions of calcium mineral deposition **(B)** and expressions of IBSP and BGLAP **(C)**. *P < 0.05 compared with the pH = 7.0 group; ^**#**^*P* < 0.05 compared with the pH = 7.4 group. Western blotting showing that GPR4 inhibition blocked the proton-induced cytoplasmic translocation of YAP **(D)** and phosphorylation of YAP and LATS1 **(E)** in the BMSCs-derived OBs. **(F)** YAP localization was assessed by immunofluorescence assay after GPR4 interference. Scale bar: 50 μm.

It has been previously demonstrated that the proton-sensing GPR4 and GPR68 can activate and inhibit the Hippo pathway kinases LATS1/2, thereby inhibiting and activating YAP in low-pH conditions, respectively^18^,^19^. Given the different responses of the YAP target genes between the BMSCs and their derived OBs in response to increases in proton concentration, we hypothesized that GPR4 and GPR68 might be differentially expressed between BMSCs and their derived OBs. This hypothesis was confirmed by the results of qRT-PCR detection. As illustrated in Fig. 3A, GPR68 was highly expressed in undifferentiated BMSCs and markedly suppressed in their derived OBs, whereas the level of GPR4 was profoundly up-regulated during the osteogenic differentiation of BMSCs, which suggests that GPR4 might be the key signaling molecule that mediated the proton-induced osteogenesis suppression and YAP inhibition.

To determine the role of GPR4 in the pH-dependent regulation of BMSC osteogenesis, two GPR4 shRNAs (shGPR4 #1 and shGPR4 #2) were used to knockdown GPR4 in the BMSCs, and GPR4 expression was markedly decreased as measured by qRT-PCR (Fig. S4A). After 21 days of induction in osteogenic media with different pHs, alizarin red S staining and qRT-PCR analyses were performed on the induced cells. The results revealed that the low pH-induced inhibitory effects on calcium mineral deposition and expressions of BGLAP and IBSP were dramatically blocked (Fig. 3B,C), which suggested that GPR4 expression was essential for the proton-induced inhibition of BMSC osteogenesis.

Subsequently, we explored the role of GPR4 in pH-dependent YAP regulation. After inducing BMSCs for 7 or 21 days, the cells were transferred to serum-free basal culture media for 24 hours and then incubated in basal culture media with different pHs for an additional three hours. Nuclear/cytosolic fractionation assay and western blotting was then performed (Fig. 3D,E and Fig. S4B,C). The results revealed that a decrease in the extracellular pH induced the cytoplasmic sequestration of YAP and enhanced the levels of phosphorylated YAP (p-YAP) and p-LATS1 in the BMSCs-derived OBs, indicating that low pH leads to LATS1 activation and YAP inhibition. However, these effects induced by proton were significantly abolished by GPR4 inhibition. YAP localization was also assessed by immunofluorescence assay after GPR4 interference (Fig. 3F). The result showed that the silencing of GPR4 blocked the cytoplasmic localization and inactivation of YAP in low pH conditions, which was consistent with the data of western blotting. All of these results suggested that the proton-induced inhibition of BMSC osteogenesis was mediated through the GPR4-induced activation of LATS1 and subsequent inhibition of YAP. Additionally, we found that in the undifferentiated BMSCs, protons exerted the opposite effect on YAP activity; i.e., lower pH conditions facilitated the nuclear translocation of YAP (Fig. S5), which might have resulted from the low expression of GPR4 and the high level of GPR68 in BMSCs.

### cAMP/PKA was required for the pH-dependent regulation of osteogenesis and YAP activity

**Figure 4:**
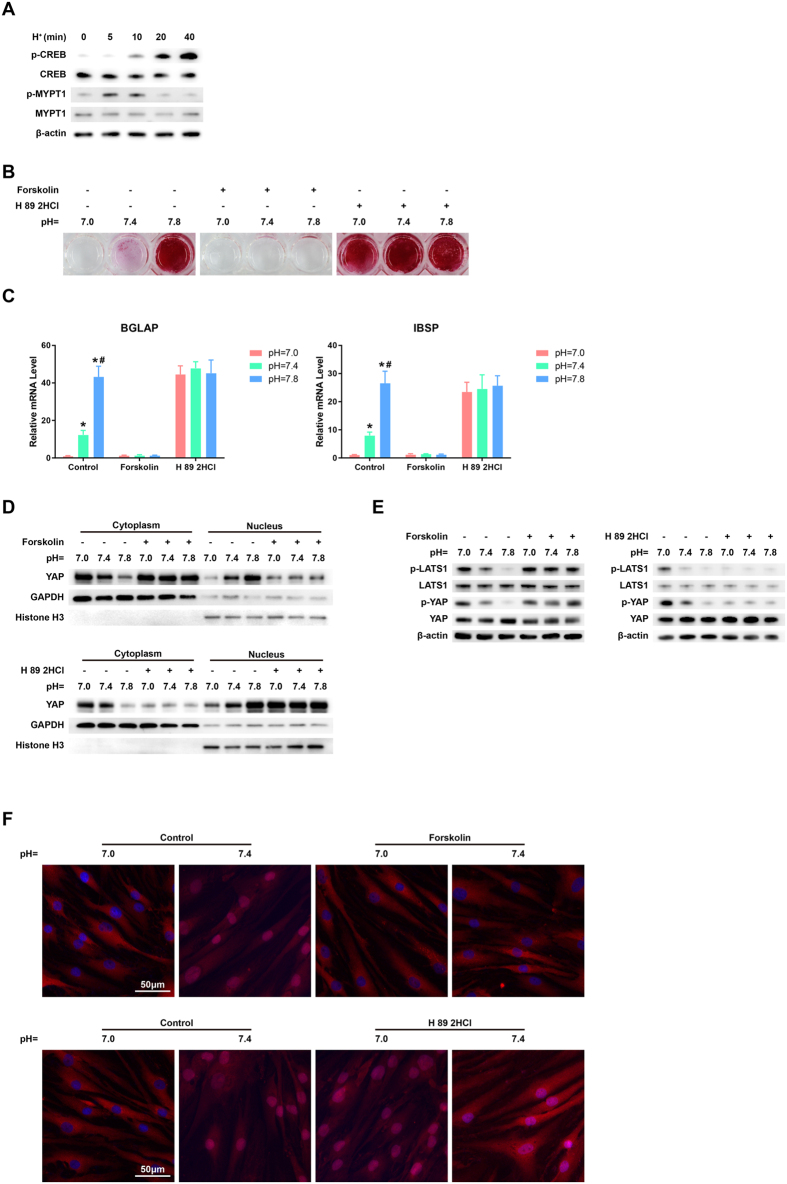
cAMP/PKA is required for the pH-dependent regulation of osteogenesis and YAP activity. BMSCs were incubated in osteogenic media for 21 days and then transferred to low pH (pH = 7.0) basal media for different times (t = 0, 5, 10, 20, or 40 min). The phosphorylation of MYPT1 and CREB was assessed by western blotting **(A)**. BMSCs were incubated in osteogenic media at different pHs containing a cAMP activator (forskolin) or a PKA inhibitor (H 89 2 HCl) for 21 days. The calcium deposits in the differentiated BMSCs were evaluated by Alizarin red S staining **(B)**, and the expressions of IBSP and BGLAP were examined by qRT-PCR analysis **(C).** *P < 0.05 compared with the pH = 7.0 group; ^**#**^*P* < 0.05 compared with the pH = 7.4 group. **(D,E)** The pH-dependent regulation of the nucleo-cytoplasmic translocation of YAP and phosphorylation of YAP and LATS1 was regulated by Forskolin and H 89 2HCl, as assessed by western blotting. **(F)** YAP localization was assessed by immunofluorescence assay after Forskolin or H 89 2HCl treatment. Scale bar: 50 μm.

The proton-sensing receptor GPR4 can induce the activation of RhoA/ROCK pathway and cAMP/PKA pathway via G_12/13_ and Gs proteins, respectively^16^,^25^, and thereby respectively inhibit and promote the kinase activities of LATS1/2 to induce YAP activation and inactivation^18^. To determine which signaling in BMSCs-derived OBs was induced by low pH, the cells were cultured in low pH (pH = 7.0) basal media for different time periods (t = 0, 5, 10, 20, or 40 min) and western blotting was performed to assess the levels of MYPT1 (the downstream target of G_12/13_/RhoA/ROCK signaling) and CREB (a representative target of Gs/cAMP/PKA pathway). The result showed that MYPT1 phosphorylation in BMSCs-derived OBs was induced by low pH at the very early phase (t = 5 min), but inhibited with the time prolonging; The level of phosphorylated CREB (p-CREB) was enhanced after the cells were incubated in low pH media for 10 min, and increased gradually with prolonged time (Fig. 4A and Fig. S6A). Our datum indicated that Gs/cAMP/PKA signaling was the critical pathway that induced by the low pH conditions. As cAMP/PKA pathway can inhibit the activity of RhoA/ROCK signaling and thereby prevent the phosphorylation of its downstream target MYPT1 [3, 4, 5, 6, 7], the mechanism of the rapid inactivation of MYPT1 might be due to the inhibitory effect of Gs/cAMP/PKA signaling.

**Figure 5:**
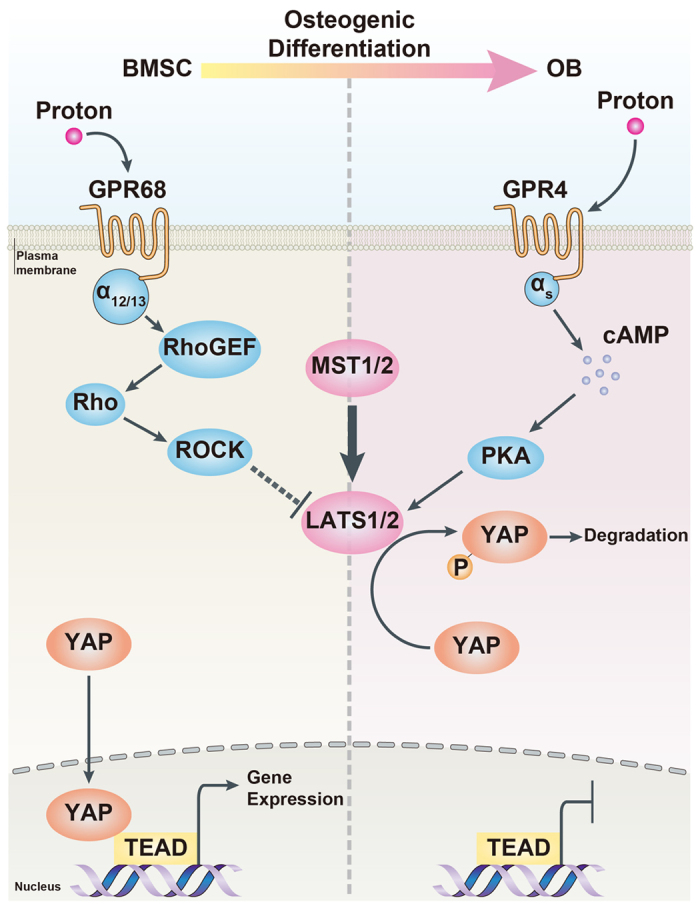
A proposed model for the functions of proton-sensing GPCRs and G proteins in the regulation of YAP activity and subsequent osteogenic differentiation of BMSCs.

To explore the role of cAMP/PKA in the pH-dependent regulation of osteogenesis and YAP activity, we treated BMSCs with forskolin, which is an activator of adenylyl cyclase that results in cAMP production. After osteogenic induction for 21 days, alizarin red S staining and qRT-PCR were performed. We found that forskolin effectively enhanced the acidic pH-induced inhibitory effects on mineralized nodule formation and the expressions of BGLAP and IBSP (Fig. 4B,C). However, after the cells were treated with the selective cAMP-dependent PKA inhibitor (H89), the proton-induced inhibition of BMSC osteogenesis was markedly abrogated. Moreover, by using western blotting (Fig. 4D,E and Fig. S6B,E) and immunofluorescence assay (Fig. 4F), we found that forskolin stimulation induced the phosphorylation of LATS1 and YAP and promoted the cytoplasmic accumulation of YAP in response to the reduction in proton concentration, whereas H89 treatment resulted in the dephosphorylation of LATS1 and YAP and enhanced the nuclear localization of YAP. Our findings suggested that the proton-induced inhibitory effects on osteogenesis and YAP activity were mediated by the activation of GPR4/cAMP/PKA signaling. A schematic diagram of the function of the proton-sensing GPCRs and G proteins in the regulation of the Hippo-YAP pathway and subsequent osteogenic differentiation of BMSCs is provided in Fig. 5.

## Discussion

Acidic microenvironments created by the accumulation of lactate and protons often accompany many pathological circumstances. Under ischemic, inflammatory, and tumorous conditions, anaerobic glycolysis is induced by hypoxia and causes lactate production, which leads to extracellular acidification^26^. Acidic pHs have been reported to exert profound effects on immune cell function^3^, cancer cell migration^27^, and the angiogenic responses of endothelial cells^28^. More recently, we found that decreases in extracellular pH enhance the proliferative and anti-apoptotic responses of BMSCs^19^. In the present study, we demonstrated that a reduction in the extracellular pH inhibited the osteogenic differentiation of BMSCs.

Decreases in osteogenesis contribute to the development and progression of various bone diseases, such as osteomyelitis, osteonecrosis, and cancers that have metastasized to the bones^7^,^8^,^9^. Inflammatory, ischemic, or tumorous conditions might induce acidic microenvironments, which would result in the further inhibition of osteogenesis as indicated by our data. Thus, it is urgent for us to study the underlying proton-sensing mechanisms to break this vicious circle.

Proton-sensing GPCRs are expressed in a wide range of cell types and can sense extracellular protons and thereby stimulate a variety of cellular activities through several types of G proteins^16^. GPCR signaling is rather complicated due to the expression of multiple receptors for protons and the differential functions of G proteins^19^. Our data revealed that GPR4 and GPR68 were differentially expressed between BMSCs and their derived OBs. In the undifferentiated BMSCs, the expression of GPR4 was inhibited, but GPR68 was highly expressed. After differentiation into OBs, GPR4 expression was markedly enhanced, whereas GPR68 was suppressed. The results of the osteogenesis-related assays revealed that GPR4/cAMP/PKA signaling mediated the proton-induced inhibitory effects on BMSC osteogenesis because the effects could be abolished by shGPR4 or the PKA inhibitor and intensified by the cAMP activator. Moreover, we identified YAP, which is an oncoprotein that promotes the osteogenic differentiation of BMSCs^29^, as the downstream effector of proton-sensing GPR4/cAMP/PKA signaling. We found that YAP activity was negatively regulated by this signaling pathway and that the overexpression of YAP effectively abrogated the osteogenesis inhibiting effects of the excess protons. Additionally, our previous work demonstrated that YAP can be activated by GPR68-Rho signaling in acidic conditions and increase the proliferation and anti-apoptotic abilities of BMSCs^19^. Our findings suggest that the regulation of GPCRs-YAP signaling may be a novel and potent strategy for improving BMSC function in acidic microenvironments, which would be beneficial in the treatment of some bone diseases that involve acidic conditions.

In addition to regulating the activities of BMSCs, acidic pHs can also influence the biological functions of other types of cells, such as immune cells^3^, tumor cells^27^, and vascular cells^28^. It is possible that the extracellular pH may also regulate the behaviors of these cells through proton-sensing GPCRs-YAP signaling. However, future investigations are needed to thoroughly address these important biological issues.

## Methods

### Cell preparation and culture

This study was approved by the Institutional Ethics Review Committee at Shanghai Sixth People’s Hospital. An informed consent was obtained from all subjects. All experiments on human BMSCs and OBs were carried out in accordance with approved guidelines and regulations. Briefly, trabecular bone was obtained during surgery from donors who required amputation due to severe trauma. The trabecular bone was transferred to a 25-cm^2^ culture flask. The BMSCs were isolated by the adherence separation method. Primary human OBs were obtained by collagenase digestion of the cortical bone from the donors. The cells were cultured in α-MEM medium (Hyclone, Logan, UT, USA) containing 10% fetal bovine serum (FBS; Gibco, Grand Island, NY, USA) and 100 U/mL penicillin and streptomycin (Gibco) at 37 °C in 5% CO_2_ in a humidified environment. The non-adherent cells were removed, and the adherent cells were passaged after becoming 80% confluent.

### Osteogenesis induction

The BMSCs were seeded into 6- or 48-well plates (1 × 10^4^ cells/cm^2^) and cultured in α-MEM medium for 24 hours. Next, the cells were rinsed with phosphate-buffered saline (PBS), and the medium was replaced with osteogenic differentiation media (StemPro osteogenesis differentiation kit; Invitrogen, USA) with different pHs. Half of the medium was changed every 2 days. Alizarin red S staining, gene expression analysis, and western blotting was performed at the indicated times.

### Alizarin red S staining

After induction with osteogenic media for 21 days, the cells were washed with PBS, fixed with 4% paraformaldehyde for 10 minutes, and incubated with 2% alizarin red S solution (Sigma, St. Louis, Mo, USA) for 30 min at 20 °C according to the manufacturer’s protocol. After washing with distilled water, the stained cells were examined using an inverted microscope (Leica DMI6000B, Solms, Germany).

### Immunofluorescence staining

After inducing BMSCs for 7 days, the cells were transferred to serum-free basal culture media for 24 hours and then incubated in basal culture media with different pHs for an additional three hours. Cells were washed with PBS and fixed with 4% paraformaldehyde for 15 min, permeabilized with 0.1% Triton X-100 for 10 min, and blocked with 3% BSA for one hour. Cells were incubated with the rabbit anti-human YAP antibody (Cell Signaling Technology, Danvers, MA, USA) for 2 hours and the Alexa Fluor^®^594-conjugated goat anti-rabbit IgG secondary antibody (Cell Signaling Technology) for 1 hour at room temperature. Irrelevant isotype-matched antibodies were used as negative controls. Nuclei was stained with 4, 6-diamidino-2-phenylindole (DAPI; 0.5 μg/ml; Invitrogen, Grand Island, NY) for 5 min. Cells were analyzed with a fluorescence microscope (Leica DMI6000B, Germany).

### Quantitative real-time PCR (qRT-PCR)

Total RNA was extracted using TRIzol Reagent (Invitrogen, Carlsbad, USA), and cDNA was synthesized from 1 μg of total RNA using the TransScript All-in-One First-Strand cDNA Synthesis SuperMix (Transgen Biotech, Beijing, China). Next, qRT-PCR was performed using FastStart Universal SYBR Green Master Mix (Roche, Indianapolis, IN, USA) with an ABI PRISM^®^7900HT System (Applied Biosystems, Forster City, CA, USA). Beta-actin was used for the normalization of the results. The primers used in this study were the following:

IBSP-FOR: GGCAGTAGTGACTCATCCGA

IBSP-REV: AGTGTGGTATTCTCAGCCTCA

BGLAP-FOR: CACCGAGACACCATGAGAGC

BGLAP-REV: CTGCTTGGACACAAAGGCTGC

RUNX2-FOR: CTGAGATTTGTGGGCCGGAG

RUNX2-REV: CTGTCTGTGCCTTCTGGGTT

OSX-FOR: CCAGACCTCCAGAGAGGAGA

OSX-REV: GGGGACTGGAGCCATAGTGA

GPR4-FOR: TCCCCAGTTTTCCCCTCTCA

GPR4-REV: TGACCAGTGACACACCAACC

GPR65-FOR: CCCCTCAGCAGTGTTGGTTT

GPR65-REV: TTTCCTTCCATATTTCTTCCGGTG

GPR132-FOR: GCGGGGCCCTGGCTTTAT

GPR132-REV: TCGGTCCTATCAAGACGTTCAC

GPR68-FOR: CCTCACCTGGTTGCAGAGAC

GPR68-REV: GACCCCCACCTGTGCTTTC

CTGF-FOR: TGTGCACCGCCAAAGATGG

CTGF-REV: ACGTGCACTGGTACTTGCAG

Cyr61-FOR: AAGGAGCTGGGATTCGATGC

Cyr61-REV: CATTCCAAAAACAGGGAGCCG

ANKRD1-FOR: AGCCCAGATCGAATTCCGTG

ANKRD1-REV: TGAGCAACTTATCTCGGGCG

### Lentivirus infection and RNA interference

S127A, shGPR4#1, shYAP#1, and shYAP#2 lentiviruses were obtained from Genepharm (Shanghai, China). shCTGF was obtained from Santa Cruz Biotechnology (sc-39329; CA, USA). The shGPR4#2 plasmid was purchased from Sigma (St. Louis MO, USA), and the virus packaging was performed by GenePharma. Cells transfection was performed following the handbook from GenePharma. Briefly, the cells were incubated in retroviral supernatant with 5 μg/mL Polybrene for 24 hours. Forty-eight hours after infection, the cells were selected with 2.5 μg/mL puromycin (Sigma-Aldrich, St. Louis MO, USA) in the culture medium. The shRNA sequences used in this study were the following:

shYAP#1: 5′-CTGGTCAGAGATACTTCTTAA-3′;

shYAP#2: 5′-AAGCTTTGAGTTCTGACATCC-3′;

shGPR4#1:5′-AAAAAAGTGCTGGCGACAGCACCTTCAACTACACCCAAGCTTCGGTGCAGCTGAAGATGCTGCCGCCAGCACGGTGTTTCGTCCTTTCCACAA-3′;

shGPR4#2: Sigma SHCLND-NM_005282_.

### Inhibitor and agonist

Forskolin and H 89 2HCl (H89) were obtained from Selleck (Houston, TX, USA), and recombinant human CTGF protein was purchased from Pepro Tech Inc. (Rocky Hill, NJ, USA). The BMSCs were cultured in osteogenic medium containing the indicated concentration of forskolin (15 μM), H 89 2HCl (20 μM) or CTGF (50 ng/mL). After 21 days of osteogenic induction, alizarin red S staining, western blotting, and gene expression analyses were performed.

### Preparation of nuclear and cytosolic extracts

The stepwise separation of the nuclear and cytoplasmic extracts from the cultured cells was performed using an NE-PER Nuclear and Cytoplasmic Extraction Reagents Kit (Pierce Biotechnology, Rockford, IL, USA). The protein concentrations were determined using a BCA Protein Assay Kit (Pierce).

### Western blotting

Protein samples were diluted 1:5 with protein loading buffer (6 × Transgen Biotech, Beijing, China) and heated at 95 °C for 5 min. Protein extracts were separated with sodium dodecyl sulfate-polyacrylamide gel electrophoresis (SDS-PAGE), transferred to polyvinylidene fluoride membranes, and blocked with 5% nonfat dry milk in TBST. The membranes were incubated with primary antibodies at 4 °C overnight and with the horseradish peroxidase (HRP)-conjugated secondary antibodies at 37 °C for 1 hour. Protein bands were visualized by enhanced chemiluminescence reagent (Thermo Fisher Scientific, Waltham, MA) and imaged by a FluorChem M gel documentation system (ProteinSimple, San Jose, CA). The primary antibodies (anti-YAP, anti-p-YAP, anti-LATS1, anti-p-LATS1, anti-MYPT1, anti-p-MYPT1, anti-CREB, anti-p-CREB, anti-GAPDH, anti-α-tubulin, anti-histone H3) and secondary antibodies were obtained from Cell Signaling Technology (Danvers, MA, USA). CTGF antibody was obtained from Sigma-Aldrich (St Louis, MO, USA).

### Statistical analysis

All of the experiments were repeated three times. The data are presented as the means ± the standard deviations. The differences between multiple groups were analyzed using one-way analyses of variance (ANOVAs). Independent-sample *t* tests were used to compare the means of pairs of groups. The statistical analyses were conducted using SPSS 20.0 (IBM, Armonk, NY). *P* values < 0.05 were considered statistically significant.

## Additional Information

**How to cite this article**: Tao, S.-C. *et al.* Decreased extracellular pH inhibits osteogenesis through proton-sensing GPR4-mediated suppression of yes-associated protein. *Sci. Rep.* **6**, 26835; doi: 10.1038/srep26835 (2016).

## Supplementary Material

Supplementary Figures
