# Supplementary material for: Decreased extracellular pH inhibits osteogenesis through proton-sensing GPR4-mediated suppression of yes-associated protein
Source: Sci Rep. 2016 Jun 3;6:26835. doi: 10.1038/srep26835 (PMC4891712; doi:10.1038/srep26835)
Supplement: Supplementary Figures [file srep26835-s1.doc]

**Decreased extracellular pH inhibits osteogenesis through proton-sensing GPR4-mediated suppression of yes-associated protein**

Shi-Cong Tao^1#^, You-Shui Gao^1#^, Chun-Yuan Chen^3^, Hong-Yi Zhu^1^, Ren-Long Zhang^3^, Jun-Hui Yin^2^, Yi-Xuan Chen^1^, Yue-Lei Zhang^1^, Shang-Chun Guo^2*^, Chang-Qing Zhang^1,2*^

1. Department of Orthopedic Surgery, Shanghai Jiao Tong University Affiliated Sixth People’s Hospital, 600 Yishan Road, Shanghai 200233, China
2. Institute of Microsurgery on Extremities, Shanghai Jiao Tong University Affiliated Sixth People's Hospital, 600 Yishan Road, Shanghai 200233, China
3. Graduate School of Nanchang University, 461 Bayi Road, Nanchang 330006, China

# Co-first authors: These authors contributed equally to this work.

* Corresponding authors

**Correspondence** **to:**

Shang-Chun Guo, Ph.D.

Institute of Microsurgery on Extremities, Shanghai Jiao Tong University Affiliated Sixth People's Hospital, 600 Yishan Road, Shanghai 200233, China

Tel: +86-188-1727-6288

E-mail: [achuni@126.com](mailto:achuni@126.com)

Chang-Qing Zhang, Ph.D., M.D.

Department of Orthopedic Surgery, Shanghai Jiao Tong University Affiliated Sixth People’s Hospital, Shanghai 200233, China

E-mail: [zhangcq@sjtu.edu.cn](mailto:zhangcq@sjtu.edu.cn)

**
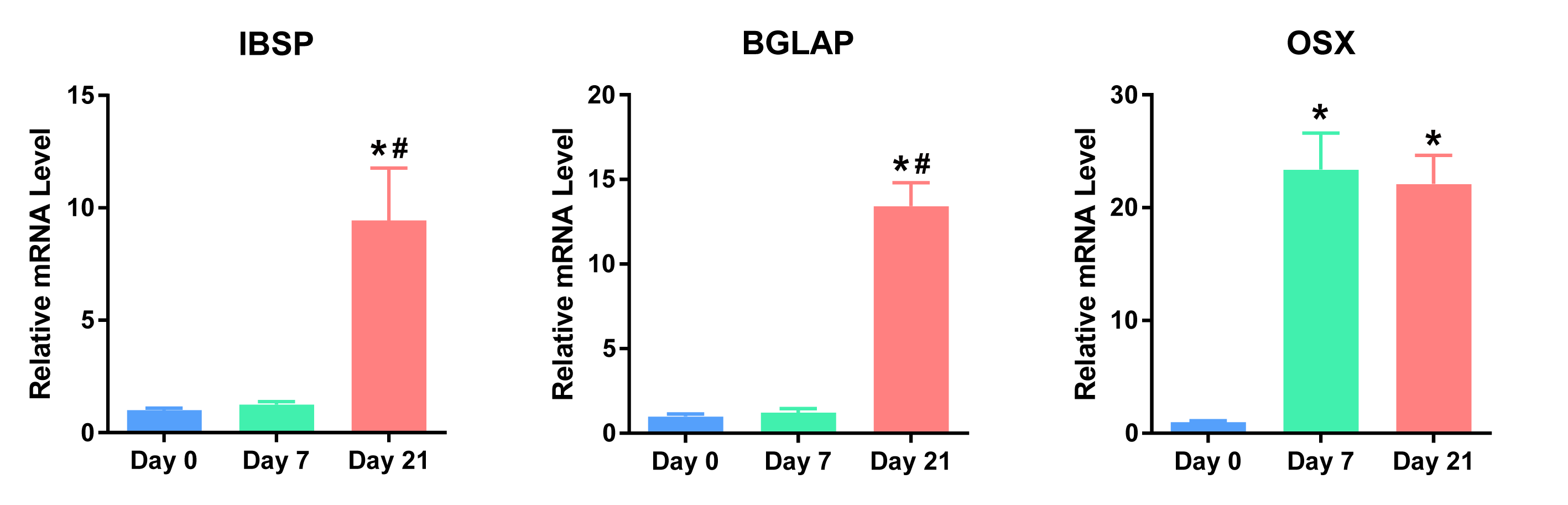
**

**Supplementary Figure S1. Expression of osteogenic-related marker genes during osteogenic differentiation of BMSCs.** The mRNA levels of BGLAP, IBSP**,** and Osx at different-stage of BMSCs’ osteogenic differentiation were detected by qRT-PCR analysis. **P* < 0.05 compared with the Day0 group; **^#^***P* < 0.05 compared with the Day7 group.

**
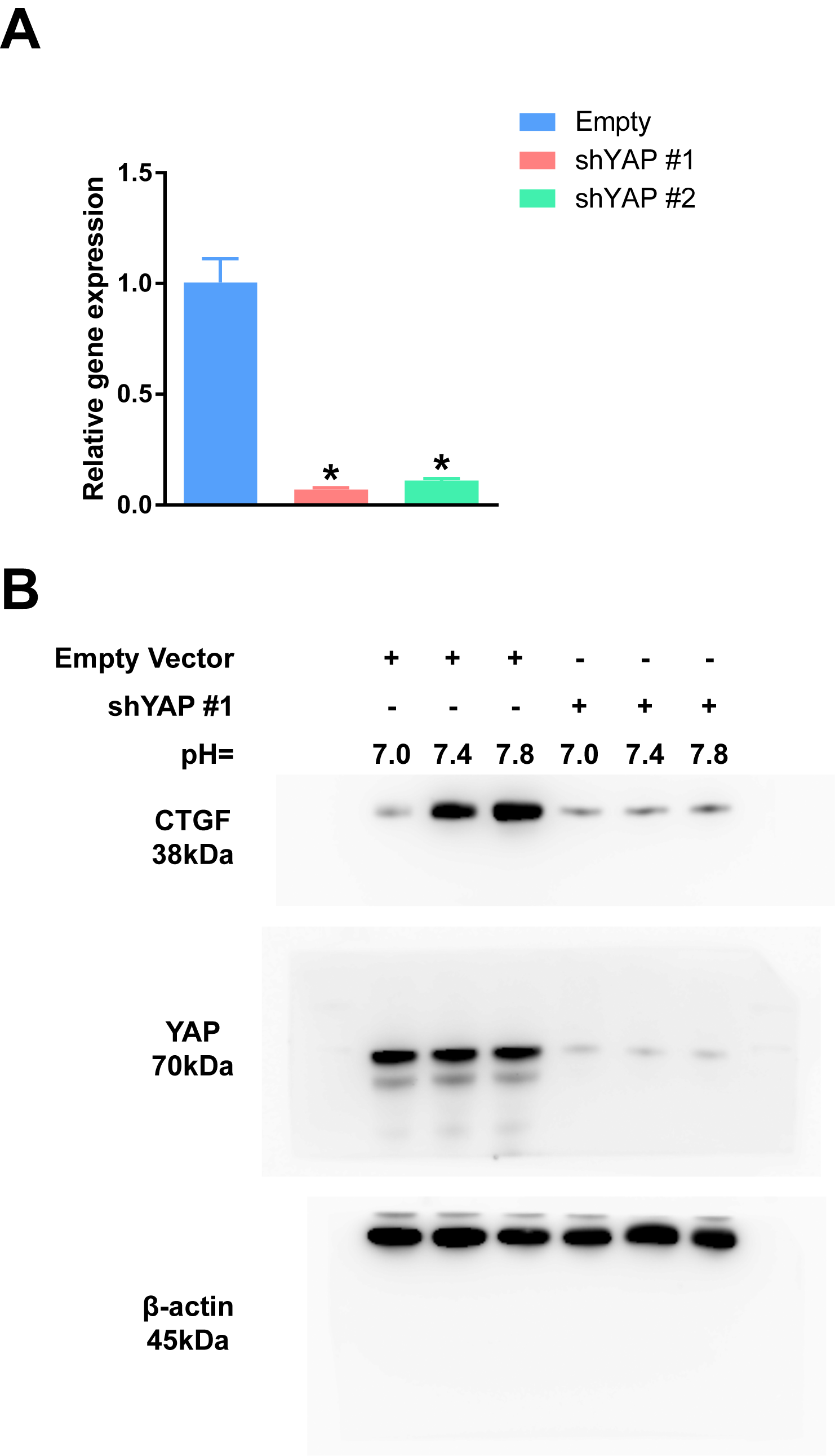
**

**Supplementary Figure S2.** **CTGF is the target gene of YAP in BMSCs-derived OBs.** (**A**) shYAP #1 and shYAP #2 were significantly inhibited the expression of YAP, as verified by qRT-PCR analysis. (**B**) The production of CTGF was markedly blocked after YAP inhibition, as determined by western blotting.

**
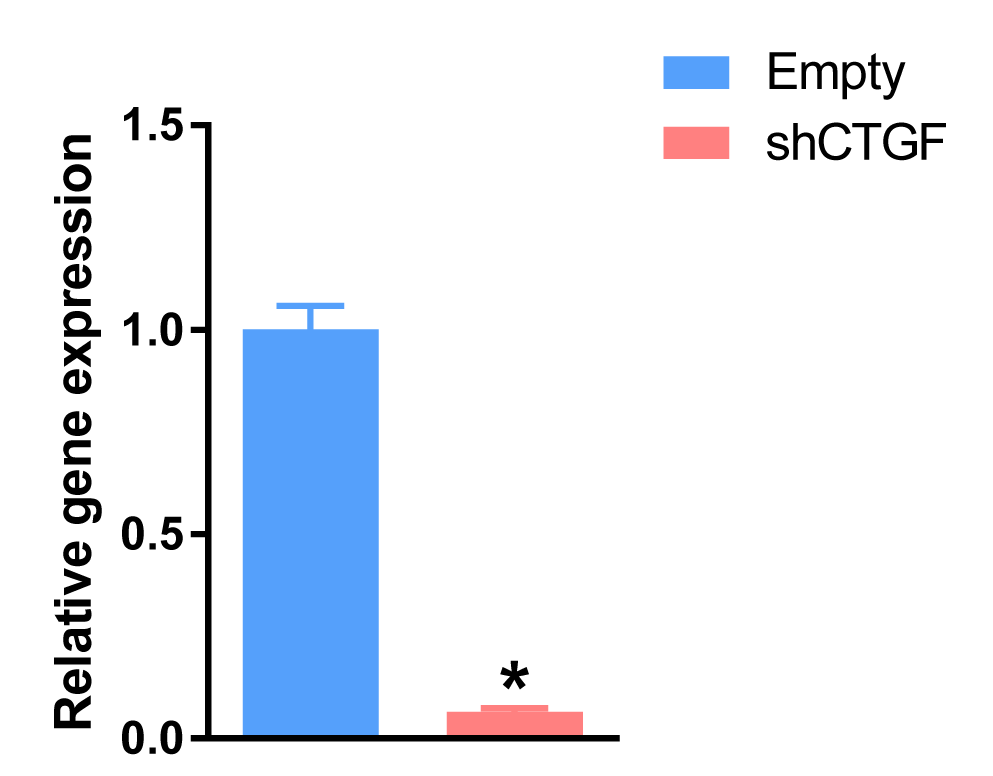
**

**Supplementary Figure S3.** The inhibitory efficiency of the shRNA for CTGF was verified by qRT-PCR. **P* < 0.05 compared with the empty vector group.

**
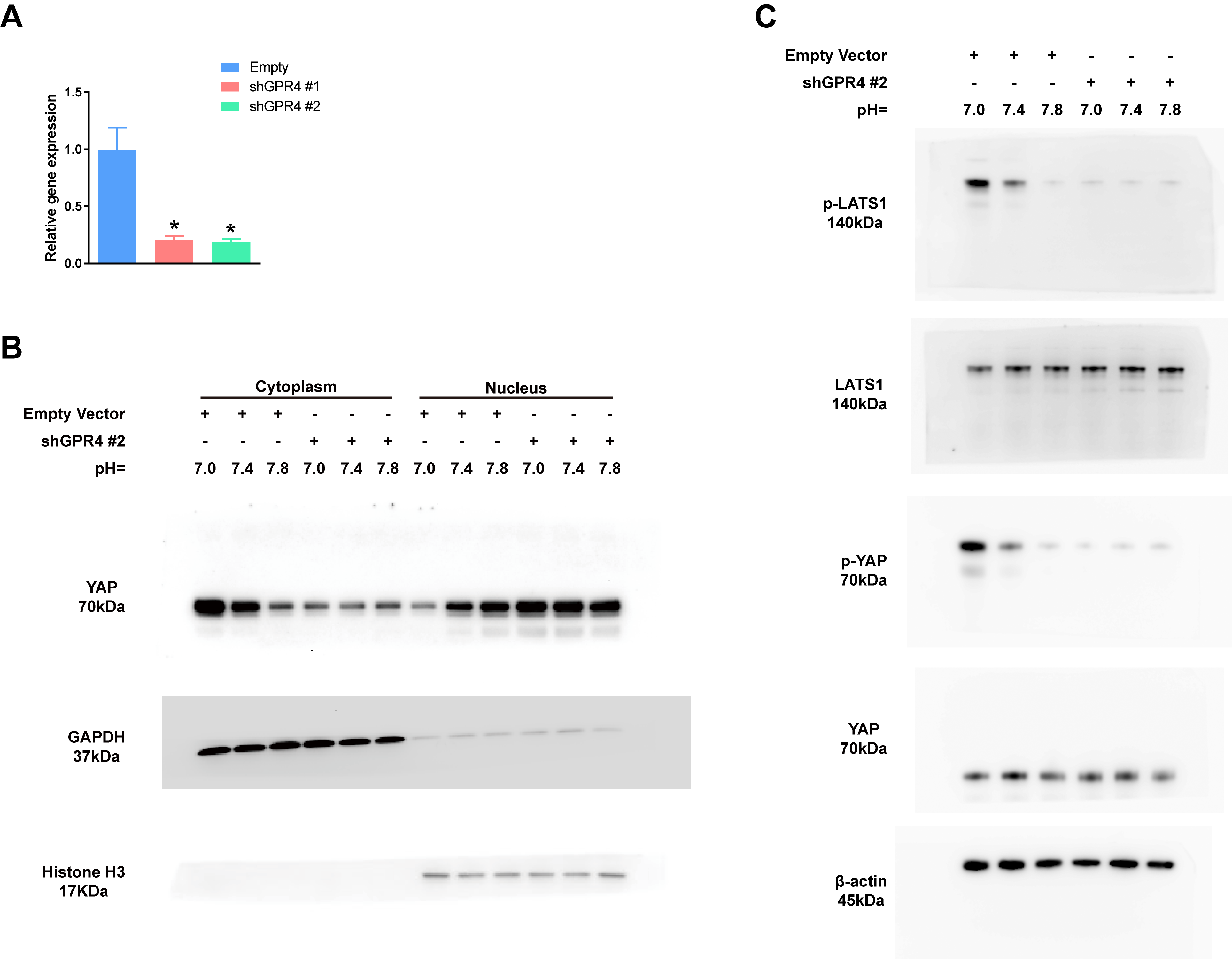
**

**Supplementary Figure S4. GPR4 mediates the proton-induced inhibition of YAP inactivation.** (**A**) shGPR4#1 and shGPR4#2 were significantly inhibited the expression of GPR4 in the BMSCs, as determined by qRT-PCR analysis. **P* < 0.05 compared with the empty vector group. (**B**) Western blotting showing that RNA interference with GPR4 abolished the proton-induced cytoplasmic translocation of YAP and maintained its nuclear localization in the BMSCs-derived OBs. (**C**) GPR4 inhibition blocked the proton-induced phosphorylation of YAP and LATS1 in the BMSCs-derived OBs, as assessed by western blotting.

**
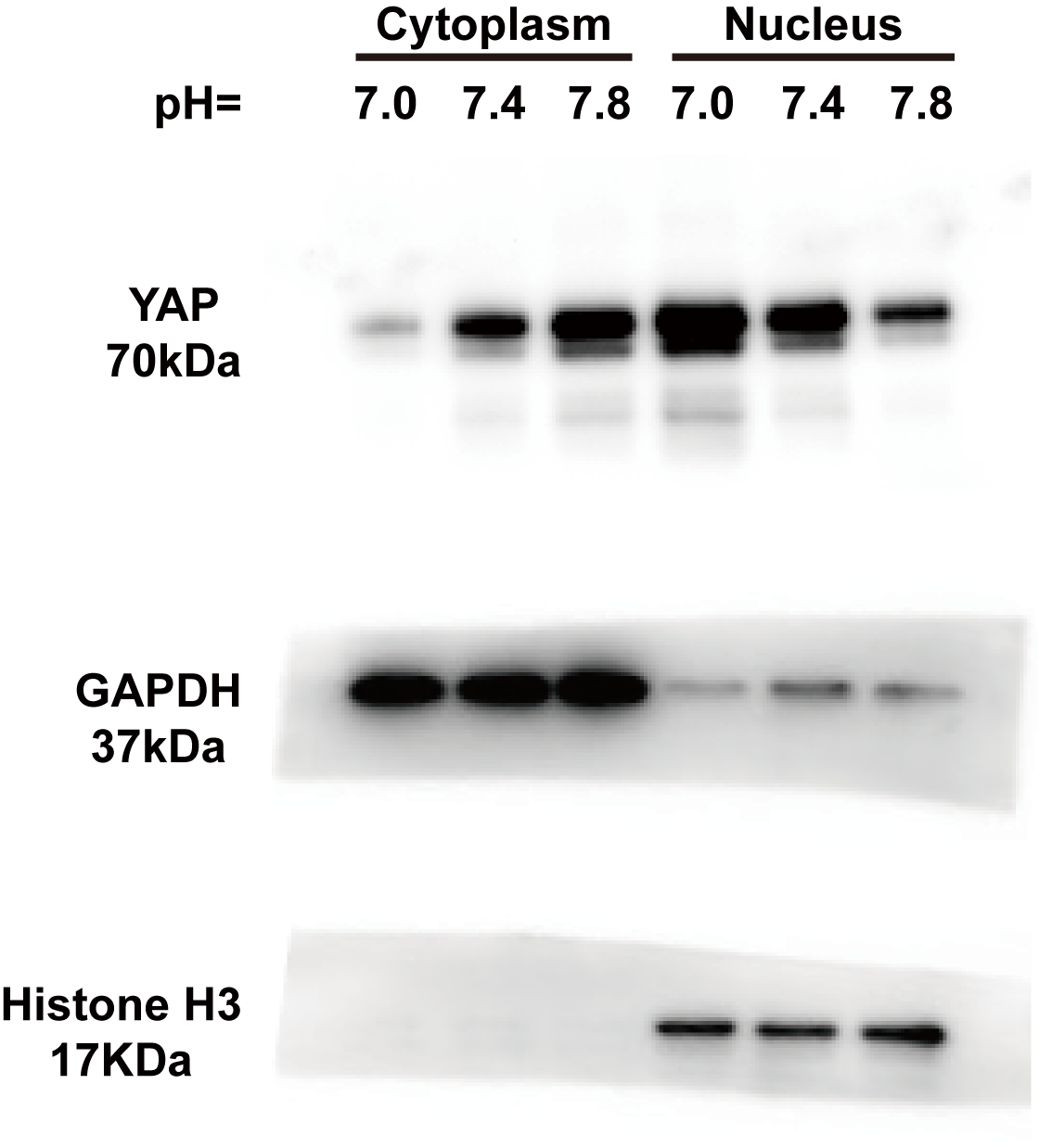
**

**Supplementary Figure S5. Low pH facilitates the nuclear translocation of YAP in the undifferentiated BMSCs.** The pH-dependent regulation of nucleo-cytoplasmic translocation of YAP was determined by western blotting.

**
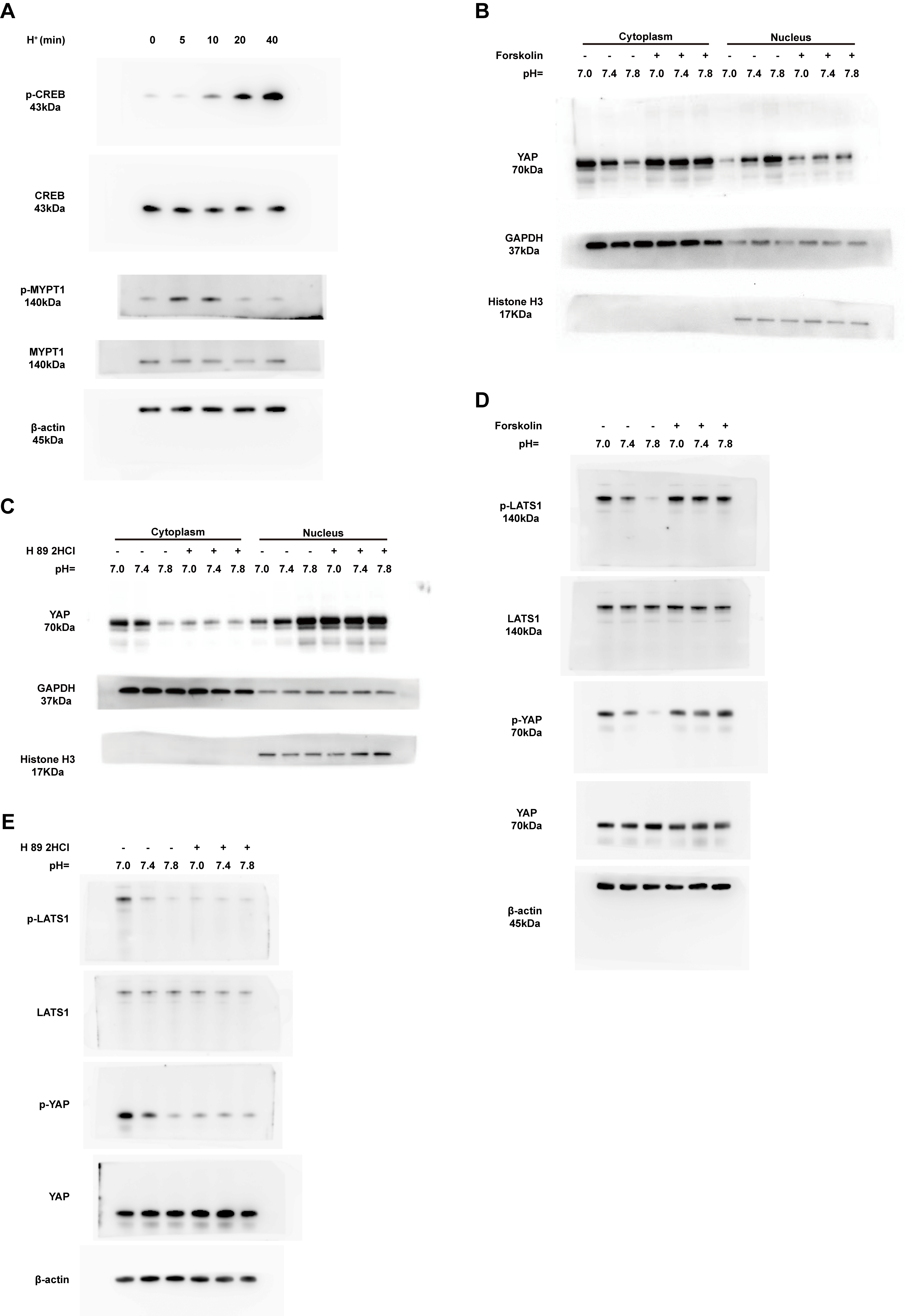
**

**Supplementary Figure S6:** Western blotting showing that, in the differentiated BMSCs, the phosphorylation of MYPT1 and CREB was regulated by extracellular pH (**A**); the pH-dependent regulation of nucleo-cytoplasmic translocation of YAP and phosphorylation of YAP and LATS1 was regulated by Forskolin (**B, D**) and H 89 2HCl (**C, E**)
